# Phantom and clinical evaluation of the Bayesian penalised likelihood reconstruction algorithm Q.Clear without PSF correction in amyloid PET images

**DOI:** 10.1186/s40658-024-00641-3

**Published:** 2024-04-22

**Authors:** Kei Wagatsuma, Muneyuki Sakata, Kenta Miwa, Yumi Hamano, Hirofumi Kawakami, Yuto Kamitaka, Tensho Yamao, Noriaki Miyaji, Kenji Ishibashi, Tetsuro Tago, Jun Toyohara, Kenji Ishii

**Affiliations:** 1 School of Allied Health Sciences, Kitasato University, 1-15-1 Kitazato, Minami-ku, Sagamihara, Kanagawa 252-0373 Japan; 2 Research Team for Neuroimaging, Tokyo Metropolitan Institute for Geriatrics and Gerontology, 35-2, Sakae-cho, Itabashi-ku, Tokyo 173-0015 Japan; 3 Department of Radiological Sciences, School of Health Sciences, Fukushima Medical University, 10-6 Sakaemachi, Fukushima-shi, Fukushima 960-8516 Japan; 4 GE HealthCare Japan, 4-7-127 Asahigaoka, Hino-shi, Tokyo 191-8503 Japan

**Keywords:** Amyloid imaging, Alzheimer’s disease, Dementia, Quantitative analysis, Regularised reconstruction

## Abstract

**Purpose:**

Bayesian penalised likelihood (BPL) reconstruction, which incorporates point-spread-function (PSF) correction, provides higher signal-to-noise ratios and more accurate quantitation than conventional ordered subset expectation maximization (OSEM) reconstruction. However, applying PSF correction to brain PET imaging is controversial due to Gibbs artefacts that manifest as unpredicted cortical uptake enhancement. The present study aimed to validate whether BPL without PSF would be useful for amyloid PET imaging.

**Methods:**

Images were acquired from Hoffman 3D brain and cylindrical phantoms for phantom study and 71 patients administered with [^18^F]flutemetamol in clinical study using a Discovery MI. All images were reconstructed using OSEM, BPL with PSF correction, and BPL without PSF correction. Count profile, %contrast, recovery coefficients (RCs), and image noise were calculated from the images acquired from the phantoms. Amyloid β deposition in patients was visually assessed by two physicians and quantified based on the standardised uptake value ratio (SUVR).

**Results:**

The overestimated radioactivity in profile curves was eliminated using BPL without PSF correction. The %contrast and image noise decreased with increasing β values in phantom images. Image quality and RCs were better using BPL with, than without PSF correction or OSEM. An optimal β value of 600 was determined for BPL without PSF correction. Visual evaluation almost agreed perfectly (κ = 0.91–0.97), without depending on reconstruction methods. Composite SUVRs did not significantly differ between reconstruction methods.

**Conclusion:**

Gibbs artefacts disappeared from phantom images using the BPL without PSF correction. Visual and quantitative evaluation of [^18^F]flutemetamol imaging was independent of the reconstruction method. The BPL without PSF correction could be the standard reconstruction method for amyloid PET imaging, despite being qualitatively inferior to BPL with PSF correction for [^18^F]flutemetamol amyloid PET imaging.

**Supplementary Information:**

The online version contains supplementary material available at 10.1186/s40658-024-00641-3.

## Introduction

Radiopharmaceuticals used for amyloid positron emission tomography (PET) imaging selectively and specifically bind to amyloid β (Aβ) plaques [1]. Amyloid PET is a minimally invasive imaging method that enables quantitative estimation of cortical Aβ plaque deposition, an essential element in evaluating neuropathological changes in patients with Alzheimer disease (AD) [2]. The fluorine-18-labeled amyloid tracer [^18^F]flutemetamol, has recently been developed by GE HealthCare (Milwaukee, WI, USA) [3]. The standards and guidelines published by the Society of Nuclear Medicine and Molecular Imaging (SNMMI) and the European Association of Nuclear Medicine (EANM) do not recommend a reconstruction algorithm or correction methods, but they do suggest pixel size and slice thickness as typical reconstruction conditions for amyloid PET imaging [4]. The Japanese Society of Nuclear Medicine (JSNM) has proposed a phantom test procedure to determine optimal reconstruction conditions and standardise amyloid PET imaging [5]. The determination process of reconstruction conditions for iterative reconstruction methods have been established for brain 2-deoxy-2-[^18^F]fluoro-d-glucose ([^18^F]FDG) and amyloid PET [6, 7].

The Bayesian penalised likelihood (BPL) reconstruction algorithm marketed as Q.Clear has been used in clinical PET/computed tomography (CT) scanners (GE HealthCare) [8, 9]. The BPL reconstruction includes a regularization factor, which allows more iteration processes without noise amplification than conventional ordered subset expectation maximization (OSEM) reconstruction [10–12]. The BPL frequently incorporates point-spread-function (PSF) modeling and time-of-flight (TOF), which results in higher signal-to-noise ratios (SNRs) and more accurate quantitation [13]. Consequently, it has improved the detection of small lesions more than OSEM reconstruction [14, 15]. A penalization factor in the BPL formula determines the global strength of regularization to control image noise in PET images. In Q.Clear, a β parameter that will be the penalization factor for reconstruction is selected from 1 to 10,000 [10, 11]. Although optimal β values for whole-body imaging using [^18^F]FDG have been reported [10, 11, 13, 16, 17], little is known about optimal β values for brain PET imaging. The optimal β values were 200 for neuro-oncological [^18^F]FDG imaging, β = 350–500 for pediatric brain [^18^F]FDG imaging, 150 or 300 for [^18^F]flutemetamol imaging, and β = 450 for carbon-11-labeled Pittsburgh compound B ([^11^C]PiB) [7, 17–19].

Correction using PSF increases the contrast and detectability of small lesions in [^18^F]FDG oncology PET imaging [20–22]. However, PSF correction can result in degraded quantitative accuracy due to Gibbs artefacts that appear as intensity overshoot along the edge of uptake [23, 24]. Gibbs artefacts can lead to the overestimation of PET tracers with global cortical uptake, such as [^18^F]FDG at the edge of gray matter (GM), and might not accurately reflect changes in cortical uptake attributed to pathological changes [25]. Therefore, PSF correction should be used carefully for quantitative brain PET imaging. The reconstruction algorithm in Q.Clear includes PSF correction, and users cannot remove it from Q.Clear on a PET console.

The PSF correction should not be applied to amyloid PET images that are evaluated based on the uptake distribution by GM. We used the PET Duetto reconstruction toolbox (GE HealthCare) to reconstruct amyloid PET images using the BPL without PSF correction. The present study aimed to validate whether the BPL reconstruction algorithm without PSF correction could be useful for amyloid PET imaging. We also aimed to determine the optimal β value using the BPL without PSF correction in [^18^F]flutemetamol images, and to optimize Q.Clear for brain PET imaging.

## Materials and methods

### PET/CT system

We used a Discovery MI (GE HealthCare) PET/CT, which comprises cerium doped lutetium-based scintillation crystal (LYSO) and SiPM-PET detectors combined with a 64-slice CT scanner. The axial and transaxial fields of view (FOV) were 20 and 70 cm, respectively, with 71 image planes spaced at 2.79-mm intervals. The timing resolution was 375 ps, and the spatial resolution according to the NEMA NU 2-2012 protocol was 3.91 mm at full width at half maximum (FWHM) [9].

### PET reconstruction condition

All data were reconstructed using the following algorithms and conditions: three-dimensional OSEM with TOF, 4 iterations, 16 subsets, and a 128 × 128 matrix; FOV, 256 mm; 2.0 mm/pixel; Gaussian filter, 4.0 mm (FWHM). These are the clinical conditions at our institution. The BPL reconstruction with TOF and with PSF correction (β = 300) was based on a previous study, and without PSF correction using β values of 50, 80, 100, 200, 300, 400, 500, 600, 700, 800, 900, and 1,000. The matrix size and FOV were set to 128 × 128 and 256 mm, respectively [18]. We reconstructed PET images using a workstation running the Duetto reconstruction toolbox for MATLAB R2017b (MathWorks Inc., Natick, MA, USA) available from GE HealthCare through a research collaboration agreement [26].

### Phantom study

#### Phantom test procedure

A Hoffman 3D brain phantom (Data Spectrum Corporation, Hillsborough, NC, USA, Hoffman phantom) and a cylindrical phantom (Itoi Plastics Co. Ltd., Kobe, Japan), each containing 20.0 MBq of [^18^F]FDG at the start of acquisition, were respectively measured for 30 min using the PET/CT in list mode. Phantom conditions and scan duration were determined according to the JSNM phantom test procedure [6]. The phantom test procedure was used to standardise the image quality of amyloid PET imaging in multi-center study of Japanese Alzheimer’s Disease Neuroimage Initiative (J-ADNI) and AMED-Preclinical study [5, 27]. Time frames of 0–170 s were extracted from every 30 min of phantom data to simulate [^18^F]flutemetamol studies and were determined based on brain radioactivity at 90 min after an injection of [^18^F]flutemetamol (185 MBq) [3].

#### Data processing

Count profiles were measured in digital and experimental images acquired from the Hoffman phantom were reconstructed using OSEM, and BPL with and without PSF correction.

Image quality was evaluated using the physical indices for phantom test proposed by the JSNM as follows: ratio of gray-to-white matter contrast calculated from images acquired from the Hoffman phantom, and image noise was calculated from those of the cylindrical phantom [6, 7].

Briefly, %contrast was calculated as:

$$\% Contrast \left( \% \right) = \frac{{\left( {GM_{p} /WM_{p} - 1} \right)}}{{\left( {GM_{d} /WM_{d} - 1} \right)}} \times 100,$$

where *GM*_*p*_ and *WM*_*p*_ are the amounts of radioactivity in GM and white matter (WM) respectively, at regions of interest (ROIs) on Hoffman phantom PET images, and *GM*_*d*_ and *WM*_*d*_ are the amounts of radioactivity of gray and white matter, respectively, at ROIs on digital Hoffman phantom images. The JSNM ROI templates are defined such that the digital Hoffman phantom provided a true gray-to-white ratio of 4 and were applied to the image co-registered to the digital phantom [5, 6]. We calculated %contrast using PETquactIE version 3.0 (Nihon Medi-Physics Co., Ltd., Tokyo, Japan) with a function designed to measure contrast.

A circular ROI (13-cm diameter) was placed on the center of the cylindrical phantom image to evaluate noise. The coefficient of variation (CV) as noise was calculated as:

$$Noise\left( \% \right) = \frac{SD}{{Mean}} \times 100,$$

where *SD* is the standard deviation of the activity within the circular ROI, and Mean is mean activity.

The ROIs for calculating noise were placed using PMOD v. 3.8 (PMOD Technologies LLC, Zurich, Switzerland).

The activity concentration of the Hoffman phantom was calculated by dividing the net phantom activity measured by a CRC-55tR dose calibrator (Capintec, Inc., Florham, NJ, USA), by the volume of the phantom (1.14 L). The recovery coefficients (RC) of GM and WM were calculated by dividing the activity concentration derived from an image by calculating %contrast, by that of the Hoffman phantom [28]. The activity concentration at WM is 25% of that at GM. The theoretical RCs of GM and WM were 1.0 and 0.25, respectively.

We adopted the reference standards of %contrast ≥ 55% and noise ≤ 15% for image quality, according to the acceptance criteria recommended by the JSNM [5, 6].

## Clinical study

### Individuals and data acquisition

Seventy-two individuals underwent [^18^F]flutemetamol PET acquisition between July 2020 and January 2021 at the Tokyo Metropolitan Institute for Geriatrics and Gerontology (TMIGG). One individual who deviated from the following [^18^F]flutemetamol PET acquisition protocol was excluded from this study. Then, we recruited 71 individuals who provided written informed consent to participate in the study, which proceeded according to the Declaration of Helsinki (2013) and was approved by the Ethics Committee at the TMIGG (Approval No. 28077). The participants were injected with 185 MBq of [^18^F]flutemetamol, then PET/CT images were acquired from 90‒110 min thereafter. Table 1 shows the characteristics of the individuals. The images were reconstructed using OSEM and BPL with (β = 300), and without (β = 600) PSF correction.

**Table 1:** Characteristics of individuals

	Total (n = 71)	Aβ negative (n = 45)	Aβ positive (n = 26)	*P* (negative vs. positive)
Age (y)	79.0 ± 4.1	78.9 ± 4.2	79.2 ± 3.9	0.893
Female (n)	50	29	21	0.183
Height (cm)	153.6 ± 8.0	154.2 ± 9.0	152.2 ± 5.4	0.384
Weight (kg)	55.4 ± 10.0	58.0 ± 10.9	50.5 ± 6.1	0.003
Injected dose (MBq)	181.0 ± 7.4	182.7 ± 7.8	177.6 ± 5.0	0.004
Uptake time (min)	90.1 ± 0.4	90.1 ± 0.3	90.3 ± 0.6	0.344

Values are shown as means ± standard deviation expected for gender

### Quantitative analysis

The clinical images reconstructed using OSEM and BPL with and without PSF correction were anatomically standardised, then quantitatively analysed using CortexID Suite (GE HealthCare)[18, 29–31]. The software provided anatomical volumes of interest (VOI) for prefrontal, anterior cingulate, posterior cingulate, parietal, lateral temporal, occipital, sensorimotor, and mesial temporal target regions in both sides. The volume of the VOI template is represented as supplemental data (Additional file 1: Table S1). The regional standardised uptake value ratio (SUVR) was the ratio of radioactivity (Bq/mL) measured in each target region to the pons as a reference region, and the composite SUVR was calculated by weight-averaging the regional SUVRs of the target regions. Relative errors for composite or regional SUVRs in BPL with and without PSF correction were calculated based on SUVRs in OSEM.

### Visual evaluation

All clinical images were assigned a new, unique identifier in random order. A neurologist and a nuclear medicine physician, who were blinded to all clinical and diagnostic information, visually assessed [^18^F]flutemetamol images that were anatomically standardised using VIZCalc (Nihon Medi-Physics Co., Ltd.)[29]. The individual’s image was anatomically standardised to the [^18^F]flutemetamol template in Montreal Neurological Institute coordinates using linear and non-linear deformation techniques. The optimal [^18^F]flutemetamol template was automatically selected as the template most similar to the individual’s image. Details of the standardisation programme were based on a previous report [32]. The assessors completed an electronic training program (GE HealthCare) before undertaking these evaluations. The brightness of the pons on the PET images was adjusted to 90% of the maximum intensity of the rainbow. All reconstructed images were classified as either Aβ negative (no significant cortical uptake of amyloid) or positive (significant cortical uptake of amyloid) using a binary scale from transaxial, sagittal, and coronal plane images. The assessments were until the assessors reached a consensus for all images.

### Statistical analyses

Data were statistically analysed using GraphPad Prism version 9 (GraphPad Software, San Diego, CA, USA). Table 1 shows that the age, weight, and injected dose did not significantly differ between those who were negative and positive for Aβ. Inter-reconstruction method agreement was evaluated using the Cohen kappa coefficient (κ). The SUVRs reconstructed under the three conditions were compared using repeated measurement one-way analysis of variance (ANOVA). Relationships among SUVRs for OSEM and BPL with and without PSF correction were calculated using Spearman correlation coefficients. Sensitivity, specificity, and areas under the receiver operating characteristic (ROC) curves (AUC) of SUVRs based on the results of visual evaluation by two physicians were calculated for each reconstructed image. The cut-off of a composite SUVR was determined from ROC curves, then the accuracy of Aβ decisions was calculated using the SUVR. Sensitivity and specificity were true positive rate and true negative rate. The accuracy was calculated as the amounts of true positive and true negative divided by all individuals. Values with *p* < 0.05 were considered statistically significant.

## Results

### Phantom study

**Fig. 1:**
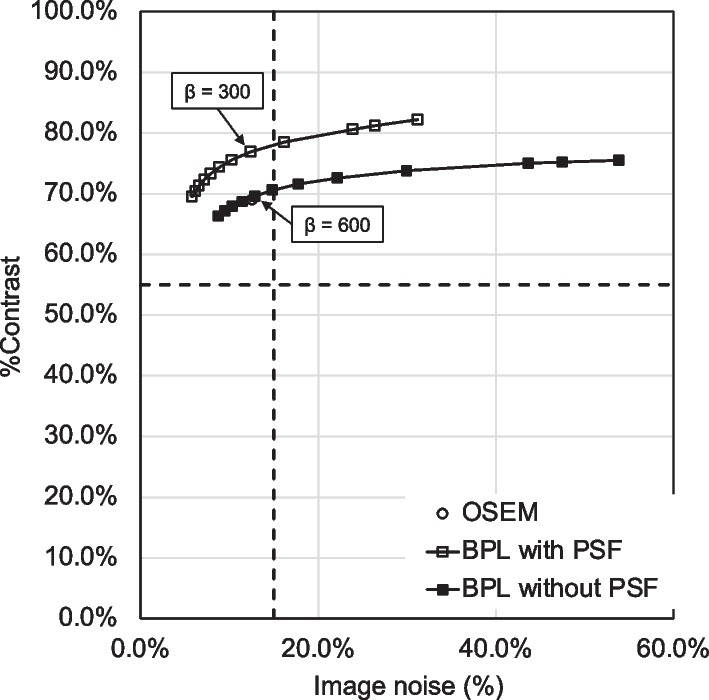
Relationship between %contrast and image noise using different reconstruction conditions. OSEM (circles), BPL with (white squares) and without (black squares) PSF correction. Curves for BPL with and without PSF correction run from left to right with increasing β values (β = 50–1000). Dotted lines, reference standards of contrast (horizontal) and noise (vertical) for image quality acceptance in Japanese Society of Nuclear Medicine phantom tests. BPL, Bayesian penalised likelihood; OSEM, ordered subset-expectation maximization; PSF, point-spread-function

Figure 1 shows the relationship between %contrast and image noise for OSEM and BPL with and without PSF correction as a function of β values in images simulating [^18^F]flutemetamol studies. The %contrast and image noise decreased as a function of larger β values for BPL with and without PSF correction. The JSNM criteria of %contrast ≥ 55% and image noise ≤ 15% were satisfied by OSEM, BPL with PSF correction within the range of β = 300–1000, and without PSF correction within the range of β = 500–1000. The %contrast was plotted as a function of image noise for reconstructing BPL without PSF correction, indicating that a balance is needed between increased contrast and decreased image noise. Ideally, these points should lie in the top-left corner of the graph [11]. Image quality was better in the order of BPL with and without PSF correction, followed by OSEM.

**Fig. 2:**
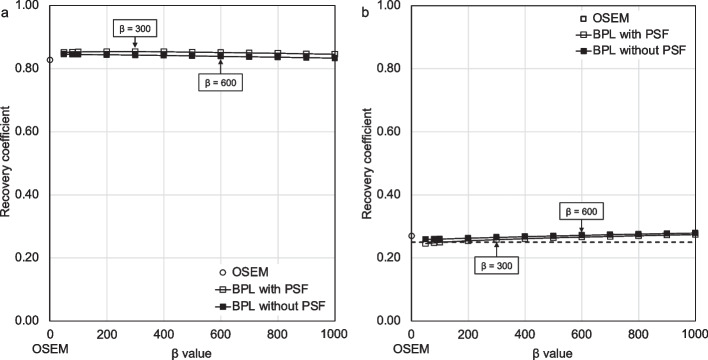
Recovery coefficients (RCs) reconstructed using OSEM and BPL with and without PSF correction. Gray matter (**a**, GM) and white matter (**b**, WM) Curves for BPL with and without PSF run from left to right with increasing β values (β = 50–1000). Theoretical RC is 1.00 at GM and 0.25 at WM (dotted line, **b**). *BPL* Bayesian penalised likelihood, *OSEM* ordered subset-expectation maximization, *PSF* point-spread-function

Figure 2 shows the RCs of GM and WM in the Hoffman phantom images reconstructed using OSEM and BPL with and without PSF correction. The RCs of GM and WM determined from the net radioactivity were respectively underestimated (1.00) and overestimated (0.25) regardless of the reconstruction algorithm. The performance of the RCs for GM and WM was better in the order of BPL with and without PSF correction followed OSEM. The optimal β value for BPL without PSF correction was taken as β = 600 based on the results of the phantom study and consultation with a physician and technologist.

**Fig. 3:**
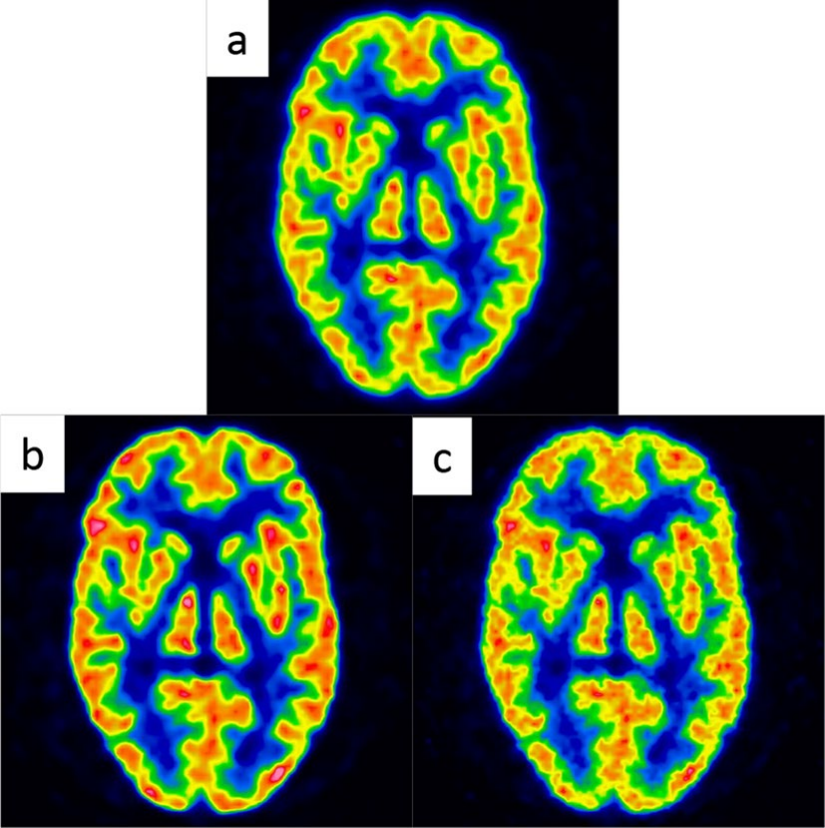
Images at Nucleus basalis level of Hoffman 3D brain phantom in three reconstruction conditions (**a**, OSEM; **b**, BPL with PSF correction; **c**, BPL without PSF correction). Images are represented 90% of maximum radioactivity. *BPL* Bayesian penalised likelihood, *OSEM* ordered subset-expectation maximization, *PSF* point-spread-function

Figure 3 shows the nucleus basalis level of the reconstructed Hoffman 3D phantom images for the three conditions. The visual impression of the phantom images supported the %contrast and image noise results. Uptake in microstructures such as the mimicked thalamus was reproduced using the BPL with PSF correction.

**Fig. 4:**
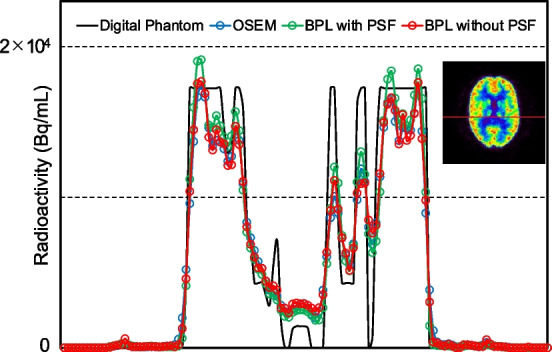
Profile curves of digital and PET images acquired from Hoffman 3D brain phantom. Red straight line for PET image acquired from the Hoffman 3D brain phantom (right) is where count profiles were measured. BPL, Bayesian penalised likelihood; OSEM, ordered subset-expectation maximization; PSF, point-spread-function

Figure 4 shows the profile curves of digital and experimental images acquired from the Hoffman phantom. The overestimated radioactivity disappeared when images were reconstructed using BPL without PSF correction.

### Clinical study

Table 2 shows Spearman correlation coefficients between SUVRs in brain regions for the three reconstruction methods. The correlation coefficients for all composite and regional SUVRs were > 0.93 between OSEM, and BPL regardless of PSF correction. The correlation coefficient between the BPL with and without PSF correction was stronger than between OSEM and the BPL without PSF correction.

**Table 2:** Spearman correlation among reconstructions

Region	OSEM versus BPL without PSF	BPL with PSF versus BPL without PSF
Composite	0.977 (0.962–0.986)	0.981 (0.969–0.988)
Prefrontal	0.960 (0.936–0.975)	0.975 (0.959–0.985)
Anterior cingulate	0.986 (0.977–0.991)	0.987 (0.979–0.992)
Precuneus	0.997 (0.996–0.998)	0.999 (0.998–0.999)
Parietal	0.994 (0.990–0.996)	0.997 (0.995–0.998)
Temporal lateral	0.991 (0.985–0.994)	0.995 (0.992–0.997)
Occipital	0.991 (0.986–0.995)	0.996 (0.994–0.998)
Sensorimotor	0.932 (0.892–0.958)	0.958 (0.932–0.974)
Temporal mesial	0.990 (0.984–0.994)	0.992 (0.988–0.995)

Values are shown as correlation coefficient (95% confidence interval)

Table 3 shows the SUVR and relative error for images of Aβ-negative and -positive participants in composite and regional brain regions reconstructed using the three methods. All SUVRs were higher for Aβ positive, than negative images. Composite and regional SUVRs did not significantly differ among reconstruction methods, except for regional SUVRs in the right and left temporal mesial regions between OSEM and BPL with PSF correction. The composite SUVR of BPL with PSF correction was slightly lower (0.01‒0.03) than the others. Relative errors for composite or regional SUVRs in BPL with and without PSF correction were < 5.0%, except for a regional SUVR of the anterior cingulate. The averaged SUV_mean_ for images of the pons from the 71 participants were respectively, 1.33, 1.34, and 1.33 for OSEM, and BPL with, and without PSF correction.

**Table 3:** Standardised uptake value ratio among reconstruction conditions

Region	Amyloid negative					Amyloid positive				
	OSEM	BPL with PSF		BPL without PSF		OSEM	BPL with PSF		BPL without PSF	
	SUVR	SUVR	Relative error (%)	SUVR	Relative error (%)	SUVR	SUVR	Relative error (%)	SUVR	Relative error (%)
Composite	0.44 ± 0.04	0.43 ± 0.04	2.3	0.44 ± 0.04	0.0	0.66 ± 0.12	0.66 ± 0.11	0.0	0.66 ± 0.12	0.0
Prefrontal R	0.40 ± 0.05	0.39 ± 0.05	2.5	0.39 ± 0.05	2.5	0.62 ± 0.14	0.62 ± 0.12	0.0	0.62 ± 0.13	0.0
Prefrontal L	0.41 ± 0.06	0.39 ± 0.06	4.9	0.40 ± 0.05	2.4	0.63 ± 0.13	0.64 ± 0.11	− 1.6	0.63 ± 0.12	0.0
Anterior cingulate R	0.44 ± 0.07	0.41 ± 0.08	6.8	0.43 ± 0.07	2.3	0.68 ± 0.14	0.66 ± 0.13	0.0	0.67 ± 0.14	1.5
Anterior cingulate L	0.48 ± 0.07	0.45 ± 0.08	6.2	0.47 ± 0.07	2.1	0.73 ± 0.14	0.72 ± 0.12	1.4	0.71 ± 0.14	2.7
Precuneus R	0.43 ± 0.05	0.41 ± 0.05	4.7	0.43 ± 0.06	0.0	0.70 ± 0.13	0.68 ± 0.12	2.9	0.68 ± 0.13	2.9
Precuneus L	0.47 ± 0.06	0.45 ± 0.06	4.3	0.46 ± 0.06	2.1	0.74 ± 0.12	0.73 ± 0.10	1.4	0.72 ± 0.12	2.7
Parietal R	0.47 ± 0.04	0.45 ± 0.04	4.3	0.46 ± 0.04	2.1	0.68 ± 0.13	0.68 ± 0.12	0.0	0.67 ± 0.12	1.5
Parietal L	0.44 ± 0.04	0.43 ± 0.04	2.3	0.44 ± 0.04	0.0	0.65 ± 0.12	0.65 ± 0.10	0.0	0.64 ± 0.12	1.5
Temporal lateral R	0.51 ± 0.03	0.49 ± 0.03	3.9	0.50 ± 0.03	2.0	0.70 ± 0.14	0.69 ± 0.13	1.4	0.69 ± 0.14	1.4
Temporal lateral L	0.48 ± 0.03	0.47 ± 0.03	2.1	0.48 ± 0.03	0.0	0.69 ± 0.12	0.69 ± 0.11	0.0	0.68 ± 0.12	1.4
Occipital R	0.50 ± 0.04	0.49 ± 0.04	2.0	0.50 ± 0.04	0.0	0.65 ± 0.12	0.64 ± 0.12	0.0	0.64 ± 0.12	1.5
Occipital L	0.50 ± 0.03	0.49 ± 0.03	2.0	0.50 ± 0.03	0.0	0.65 ± 0.12	0.65 ± 0.12	0.0	0.65 ± 0.12	0.0
Sensorimotor R	0.45 ± 0.06	0.44 ± 0.06	2.2	0.45 ± 0.06	0.0	0.55 ± 0.10	0.55 ± 0.10	0.0	0.55 ± 0.10	0.0
Sensorimotor L	0.45 ± 0.06	0.44 ± 0.06	2.2	0.44 ± 0.06	2.2	0.55 ± 0.09	0.55 ± 0.09	0.0	0.55 ± 0.09	0.0
Temporal mesial R	0.46 ± 0.04	0.44 ± 0.04	4.3	0.45 ± 0.04	2.2	0.50 ± 0.05	0.49 ± 0.05	2.0	0.50 ± 0.05	0.0
Temporal mesial L	0.46 ± 0.04	0.44 ± 0.04	4.3	0.45 ± 0.04	2.2	0.51 ± 0.06	0.50 ± 0.06	2.0	0.51 ± 0.06	0.0

Values are shown as average ± standard deviation

The Cohen kappa coefficients (95% confidence interval, CI) between two physicians were κ = 0.881 (0.767–0.994), 0.911 (0.81–1.000), and 0.882 (0.771–0.994), respectively, for OSEM, and BPL with, and without PSF correction. We considered that 29, 27, and 30 of 71 individuals were Aβ positive in OSEM, BPL with, and without PSF correction, respectively, and the Cohen kappa coefficients (95% CI) were κ = 0.941 (0.861–1.000), 0.971 (0.915–1.000), and 0.912 (0.815–1.000) for OSEM versus BPL with PSF correction, OSEM versus BPL without PSF correction, and BPL with versus without PSF correction, respectively. The visual evaluation revealed almost perfect agreement independently of the reconstruction methods.

**Fig. 5:**
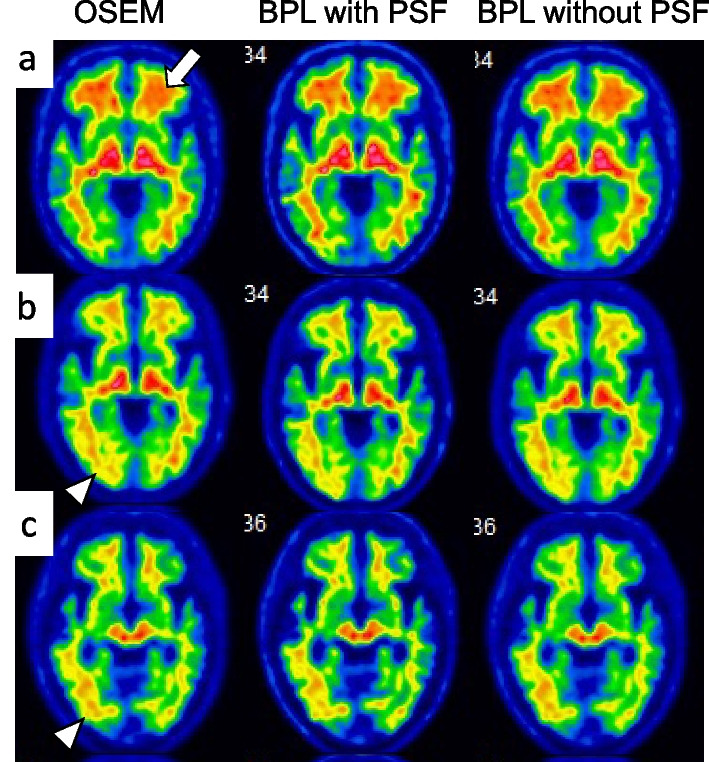
Typical false-negative (**a**) and two false-positive (**b** and **c**) images by quantitative analysis in OSEM, BPL with PSF correction, and BPL without PSF correction that disagreed. Images are from three participants. Amyloid β positive images reconstructed using BPL without PSF correction (center of **a**), and OSEM and BPL without PSF correction (both sides of **b** and **c**). *BPL* Bayesian penalised likelihood, *OSEM* ordered subset-expectation maximization, *PSF* point-spread-function

Table 4 shows the sensitivity, specificity, accuracy, AUC, and cut-off of the composite SUVRs for each reconstruction method. The composite SUVR was better when images were reconstructed by BPL with PSF correction compared with the other two methods, whereas the outcomes of the other two methods were equivalent. Rates were false-positive in 3, 1, and 3, and false-negative in 2, 1, and 2 participants for OSEM, and BPL with, and without PSF correction, respectively. Figure 5 shows anatomically standardised transverse images of three participants whose results varied from the visual findings among the three reconstruction methods. Table 5 summarizes the quantitative analysis and visual evaluation outcomes for these individuals. The composite SUVRs were 0.56, 0.54, and 0.55 for false-positive (Fig. 5a), 0.46, 0.44, and 0.46 for false-negative (Fig. 5b) images, and 0.44, 0.42, and 0.43 for one false-negative (Fig. 5c) image in OSEM, and BPL with, and without PSF correction, respectively. Three images with negative (Fig. 5a) and positive (Fig. 5b, c) Aβ deposition were classified as equivocal in visual evaluation. Another image judged as visually negative, was quantitatively classified as positive due to more abundant non-specific uptake at WM (white arrow on Fig. 5a), as the composite SUVR exceeded the cut-off value. Others who were visually positive had Aβ deposition in the right lateral temporal lobe (white arrowhead on Fig. 5b, c), but the composite SUVR was lower than the cut-off value. Visual evaluation was consequently correct in the order of OSEM, and BPL without, and with PSF correction. In addition to the above, the composite SUVR of one image reconstructed using OSEM and another reconstructed by BPL without PSF correction that were falsely judged as positive had a lower SUVR than the cut-off value due to PSF correction. The composite SUVR of the image that was falsely identified as negative did not exceed the cut-off value due to PSF correction.

**Table 4:** Sensitivity, specificity, accuracy, and area under curve of composite SUVR for each reconstruction

Reconstruction	Sensitivity	Specificity	Accuracy	Area under curve	Cut-off
OSEM	93.1	92.9	93.0	0.969	0.505
BPL with PSF	96.3	97.7	97.2	0.995	0.505
BPL without PSF	93.3	92.7	93.0	0.969	0.495

**Table 5:** Summary of outcomes of quantitative analysis and visual evaluation

Individual	OSEM		BPL with PSF correction		BPL without PSF correction	
	Quantification	Visual	Quantification	Visual	Quantification	Visual
No. 4 (Fig. 5a)	P	N	P	N	P	P
No. 42 (Fig. 5b)	N	P	N	N	N	P
No. 46 (Fig. 5c)	N	P	N	N	N	P

*N* negative, *P* positive

## Discussion

The calculation formula in the BPL reconstruction algorithm, Q.Clear includes PSF correction. However, Gibbs artefacts in brain PET images caused by PSF correction can result in abnormal distribution with enhanced cortical uptake. Therefore, PSF correction should not be applied to PET tracers that spread to the cortex. This study validated the usefulness of BPL reconstruction without PSF correction for amyloid PET imaging using [^18^F]flutemetamol. The images produced using BPL without PSF correction were artifact-free, but the image quality and quantitative values were slightly worse than those of BPL with PSF correction in the phantom study. The optimal β value for BPL without PSF correction determined in the phantom study was 600, because the image noise of BPL without (at β = 600) and with (β = 300) PSF correction were equivalent. The optimised BPL without PSF correction did not change the composite and regional SUVRs compared with the other methods. Agreement among visual evaluations of images reconstructed using the three methods was almost perfect. However, the outcomes of quantitative analysis and visual evaluations of Aβ deposition differed among the reconstruction methods and when the results were equivocal.

We argued that optimising β values of BPL according to target lesions or the tracers is important [7, 33]. Here, %contrast and image noise decreased as a function of higher β values for BPL without PSF correction. These results are consistent with our previous findings of [^18^F]FDG and [^11^C]PiB PET imaging [7]. Image quality was better in the order of BPL with and without PSF correction, followed by OSEM. The OSEM, and BPL with PSF correction at β = 300, and BPL without PSF correction at β = 500–1000 satisfied the criteria for phantom test procedures determined by the JSNM [5]. Reconstruction with PSF correction improves spatial resolution and contrast recovery and reduces spatial noise [12]. Contrast was better and image noise was reduced in images reconstructed using BPL with, than without PSF correction. We found that improving image quality with PSF correction was more effective than the relative difference penalty (RDP), which controls edge preservation and noise suppression in BPL [34, 35]. A higher β value needs to be applied to the BPL without PSF correction to suppress image noise compared with the BPL with PSF.

The performance of the RCs decreased as the β values increased in the BPL without PSF correction. The order of RCs approaching the true activity in GM and WM was BPL with and without PSF correction, followed by OSEM. The RDP function in BPL enabled more accurate quantitative value recovery than OSEM, because it allowed effective convergence in the images [10–12]. The PSF correction improved the quantity of PET images by recovering spatial resolution in the PET FOV. The establishment of harmonised brain PET image quality and quantitation has been investigated using OSEM with PSF correction in multicenter studies [28]. Our results using BPL with and without PSF correction were equivalent to and better than those of previous study in GM (0.78–0.83) and WM (0.38–0.50) using OSEM with PSF [28]. The RC performance was better in brain PET images reconstructed using BPL without PSF correction, and the best in phantom images reconstructed using BPL with PSF correction. However, Gibbs artefacts distorted the distribution of radioactivity, which might have impeded visual evaluation. One study found that PSF correction should be avoided for tracers such as [^18^F]FDG or [^11^C]flumazenil with high uptake by GM to avoid causing signal enhancement in this area [25]. We determined that β = 600 was optimal for BPL without PSF correction based on qualitative and quantitative evaluations and the visual impressions of a neurologist using [^18^F]flutemetamol images.

The composite SUVR and all regional SUVRs correlated and were stable without dependence on reconstruction algorithm or conditions. The maximum SUV determined by the maximum radioactivity using oncological [^18^F]FDG imaging studies depends on the extent of the β value in BPL due to varying image quality [16, 17, 36]. The SUVR calculated from mean radioactivity was stable within a wide range of β values [7]. Furthermore, SUVRs between reconstruction algorithms (OSEM and BPL) and reconstruction conditions (OSEM with/without PSF correction) were almost identical in [^18^F]flutemetamol images [18, 37]. The present study found that the SUVR was robust regardless of BPL reconstruction, with or without PSF correction. The results of another study that investigated various reconstruction conditions also supported our findings [38, 39]. The SUVR reconstructed by BPL with PSF correction was slightly lower than that of other methods, which corresponds to earlier findings [18]. The pons is a small anatomical structure and a hot region due to non-specific uptake for [^18^F]flutemetamol [18, 37, 40, 41]. In oncological PET imaging, PSF correction significantly enhances SUVs in small tumor lesions [12]. Therefore, the SUVR in BPL with PSF correction was lower than that of other methods. The relative error of SUVR between the OSEM or BPL without PSF correction and BPL with PSF correction was < 3.0%. The accuracy of ROC analysis in BPL with PSF correction was the highest in this study. The SUVR calculated from the pons as the reference region with PSF correction was not affected by the results of the quantitative analysis.

The inter-reader agreement rates for visual evaluation in the present study were high (κ = 0.881–0.911), and consistent with previous findings [42]. The inter-reconstruction method agreement rates were also high (κ = 0.912–0.971). Image noise increased with the BPL without PSF correction (Fig. 3). The smoothing in the anatomical standardisation process reduced image noise in VIZCalc software (Fig. 5). Therefore, the outcome of Aβ judgements determined by visual evaluation agreed not only between the two assessors but also among the reconstruction methods. Disagreement among reconstruction methods did not depend on the method, as it was due to the two assessors facing difficulties discriminating equivocal findings. Furthermore, the distribution of Aβ deposition did not substantially differ among the reconstruction methods.

The present cut-off value for composite SUVR, calculated using the pons as a reference region, was lower than in earlier studies [37, 38, 43]. This was probably due to our participants that largely comprised healthy individuals and patients with mild cognitive impairment, in which Aβ deposition is at an early stage. This is supported by a previous study that included shared individuals [29]. The results of ROC analyses were excellent regardless of reconstruction methods. The present sensitivity, specificity, and the AUC of the composite SUVR were better than in an earlier study using [^18^F]flutemetamol [37]. This was because our clinical data were less variable, stable, and generated at a single institution using an SiPM-PET system [44]. Patients with visually Aβ negative and quantitatively Aβ positive discordant images were 11% more likely to progress to AD than visually Aβ positive and quantitatively Aβ negative discordant images for SUVR with a pons reference [38]. The composite SUVR was higher in one participant (Fig. 5a) whose results were visually negative but quantitatively positive under each of OSEM and BPL with PSF correction. This was due to the anatomical volume of interest template, which collected spill-out from abundant non-specific accumulation in WM. Therefore, BPL without PSF correction, which eliminates Gibbs artefacts and is equivalent to quantitative values in OSEM, was a reasonable method for reconstructing amyloid PET images. The amyloid PET biomarker committee of the Quantitative Imaging Biomarkers Alliance (QIBA) established by the Radiological Society of North America (RSNA), also suggests that PSF correction should not be used in amyloid PET imaging [45]. In the future, if the PSF correction parameter in the BPL reconstruction algorithm is optimised, e.g. by applying hybrid space PSF correction, the BPL with modified PSF correction will mitigate edge enhancement due to Gibbs artefacts and be used for amyloid PET imaging [46].

The present study had some limitations. Images for quantitative analysis and visual evaluation were anatomically standardised without using magnetic resonance imaging (MRI). The SUVRs calculated by normalization with and without MRI normalization could discriminate Aβ deposition equally well [30]. We prioritised the replicability of our study using commercially available CortexID Suite and VIZCalc software. Here, we used the SUVR instead of the more recently introduced Centiloid scale (CL), which is now commonly used for research purposes [47, 48]. Most commercial amyloid PET image analysis software, including the CortexID Suite, does not analyze amyloid deposition levels using the CL. The CL provides a more standardised quantitative value and is more independent of scanner or tracer types. Here, we used BPL reconstruction, which is a specific reconstruction algorithm used in recently commercialised PET scanners. The evidence could be more universal if the CL is used as a quantitative value.

## Conclusions

We aimed to validate the use of BPL without PSF correction for both quality and quantity in [^18^F]flutemetamol amyloid PET images using both phantom and clinical data. We found that BPL without PSF correction corrected unnatural enhancement of cortical uptake caused by Gibbs artefacts. The image quality and quantitative values obtained from phantom images using BPL without PSF correction were respectively equivalent to, and better than those obtained using OSEM reconstruction, but worse than those obtained using BPL with PSF correction. We determined that the optimal β value for BPL without PSF correction was 600. The outcomes of visually and objectively classified Aβ deposition did not significantly differ among the three reconstruction methods. Therefore, BPL without PSF correction can be considered a new, standard reconstruction method for amyloid PET imaging.

### Supplementary Information

**Additional file 1**. **Table S1**: Volume of VOI template in CortexID Suite for amyloid imaging

## Data Availability

The datasets generated during and/or analysed during the current study are available from the corresponding author on reasonable request.
