# Supplementary material for: Phantom and clinical evaluation of the Bayesian penalised likelihood reconstruction algorithm Q.Clear without PSF correction in amyloid PET images
Source: EJNMMI Phys. 2024 Apr 22;11:37. doi: 10.1186/s40658-024-00641-3 (PMC11035535; doi:10.1186/s40658-024-00641-3)
Supplement: Supplementary file 1 — Additional file 1. Table S1: Volume of VOI template in CortexID Suite for amyloid imaging [file 40658_2024_641_MOESM1_ESM.docx]

Supplemental table 1. Volume of VOI template in CortexID Suite for amyloid imaging

| Region | Volume (mL) |
| --- | --- |
| Prefrontal R | 109.9 |
| Prefrontal L | 102.8 |
| Anterior cingulate R | 10.7 |
| Anterior cingulate L | 9.2 |
| Precuneus R | 28.9 |
| Precuneus L | 27.2 |
| Parietal R | 43.8 |
| Parietal L | 43.0 |
| Temporal lateral R | 73.4 |
| Temporal lateral L | 70.1 |
| Occipital R | 49.0 |
| Occipital L | 49.5 |
| Sensorimotor R | 23.1 |
| Sensorimotor L | 21.5 |
| Temporal mesial R | 13.4 |
| Temporal mesial L | 13.5 |
| Pons | 8.8 |

VOI, volume of interest
